# Supplementary material for: Quantum-dot-labeled synuclein seed assay identifies drugs modulating the experimental prion-like transmission
Source: Commun Biol. 2022 Jun 29;5:636. doi: 10.1038/s42003-022-03590-8 (PMC9243017; doi:10.1038/s42003-022-03590-8)
Supplement: Supplementary file 2 — Supplementary Information [file 42003_2022_3590_MOESM2_ESM.pdf]

Supplemental information

Quantum-dot-labeled synuclein seed assay identifies drugs modulating the experimental prion-like transmission

Yukio Imamura[1], Ayami Okuzumi[1,2], Saki Yoshinaga[1], Akiko Hiyama[1], Yoshiaki Furukawa[3], Tomohiro Miyasaka[4], Nobutaka Hattori[2], Nobuyuki Nukina[1] *

[1] Laboratory of Structural Neuropathology, Doshisha University Graduate School of Brain Science, 1-3 Miyakodanitatara, Kyotanabe-shi, Kyoto 610-0394, Japan

[2] Department of Neurology, Juntendo University Graduate School of Medicine, 2-1-1 Hongo, Bunkyo-ku, Tokyo 113-8421, Japan

[3] Department of Chemistry, Keio University, 3-14-1 Hiyoshi, Kohoku, Yokohama, Kanagawa 223-8522, Japan

[4] Department of Neuropathology, Faculty of Life and Medical Sciences, Doshisha University, 1-3 Miyakodanitatara, Kyotanabe-shi, Kyoto 610-0394, Japan, Kyoto, Japan

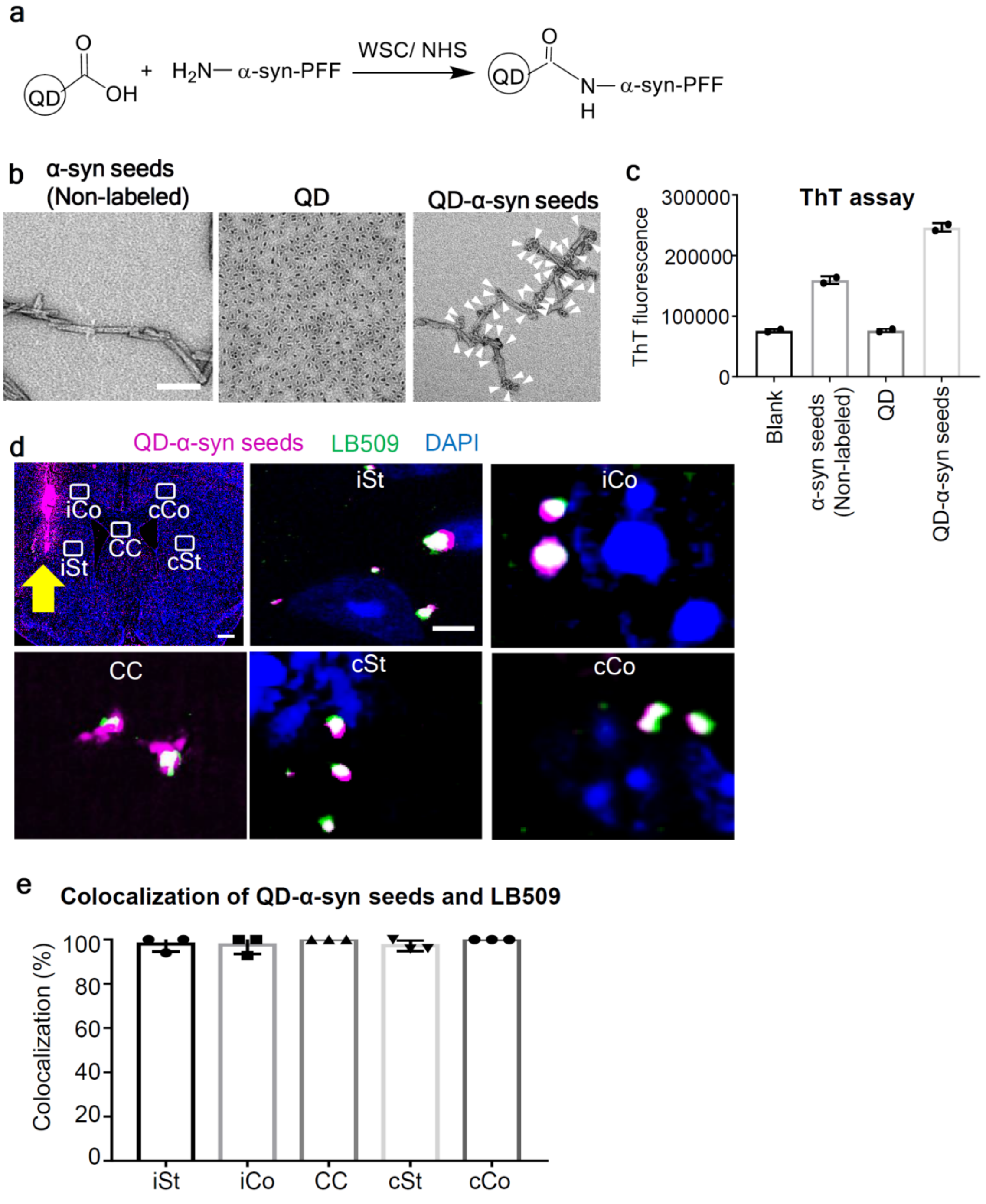

Supplemental Figure S1 α-syn-PFFs labeled by Quantum dots and their colocalization with anti-α-syn immunoreactivity in the brain

(a) QD (CdSeTe quantum dots) with carboxyl groups were reacted with amino groups of α-syn-PFFs, which directly bound to QD with covalent bond. WSC: 1-(3-Dimethylaminopropyl)-3-ethylcarbodiimide, NHS: N-hydroxysuccinimide.
(b) Transmission electron microscope image. Left: α-syn-seeds (non-labeled), middle: QD only, right: QD-α-syn seeds. White arrow head: QD-labeled. Bar: 100 nm. **(c)** thioflavin T(ThT) assay. QD-α-syn seeds and non-labeled seeds showed higher ThT fluorescence values. **(d)** Colocalization of QD-α-syn-seeds fluorescence (pink) and LB509 (α-syn immunoreactivity, green) at 1 hour after QD-α-syn-seeds injection. White: colocalization of QD-α-syn seeds and LB509 immunoreactivity. Blue: DAPI. Region of interest (ROI): iSt., ipsilateral Striatum. iCo, ipsilateral Cortex. CC, Corpus Callosum. cSt, contralateral Striatum. cCo, contralateral Cortex. Bar: slice, 500μm, ROI, 5μm. (**e**) Colocalization rates of QD-α-syn seeds and LB509 immunoreactivity in the ROIs. n = 3 mice.

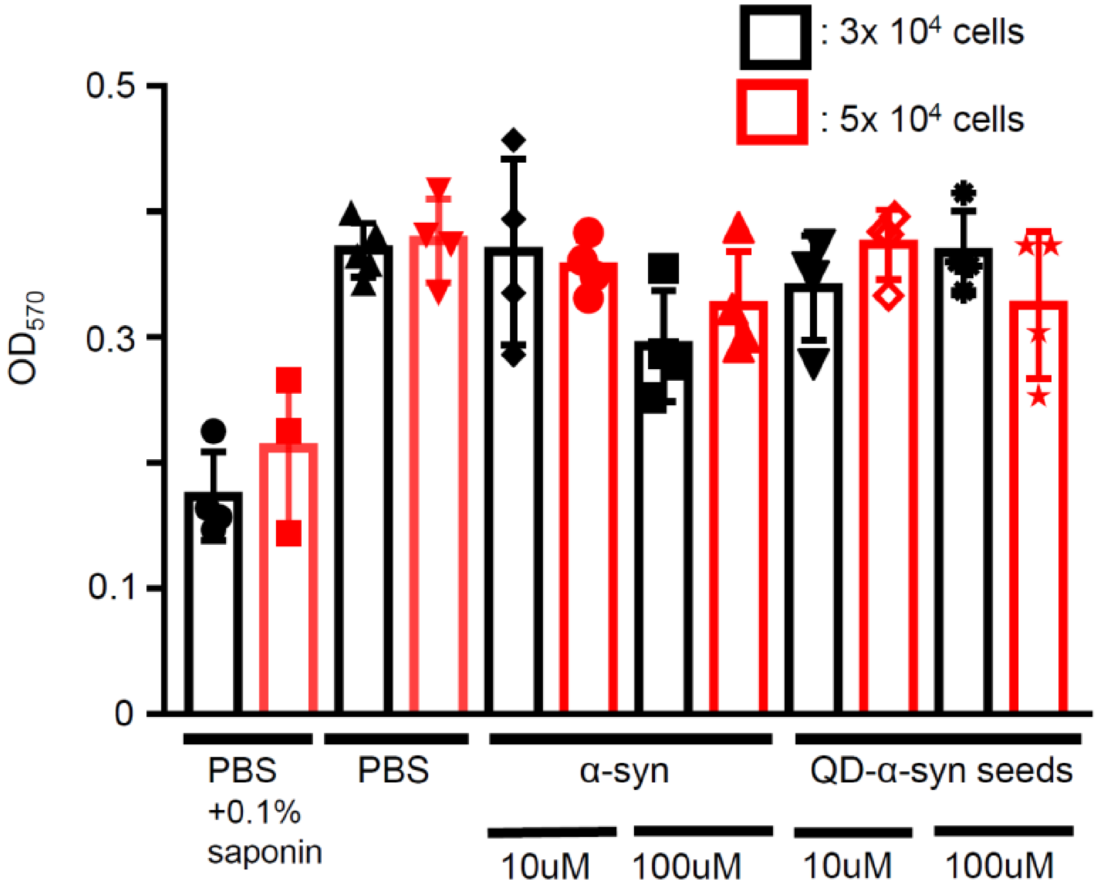

Supplemental Figure S2 No QD-α-syn seed toxicity by MTT assay in neuronal cells
OD_{570} was measured for MTT assay. No cytotoxic effect by α-syn seeds or QD-α-syn seeds treatment was observed. A line of experiments was repeatedly 3 times. Statistical differences (except PBS+0.1%saponin) were determined by one-way ANOVA and Tukey's post test. $p > 0.05$: against PBS.

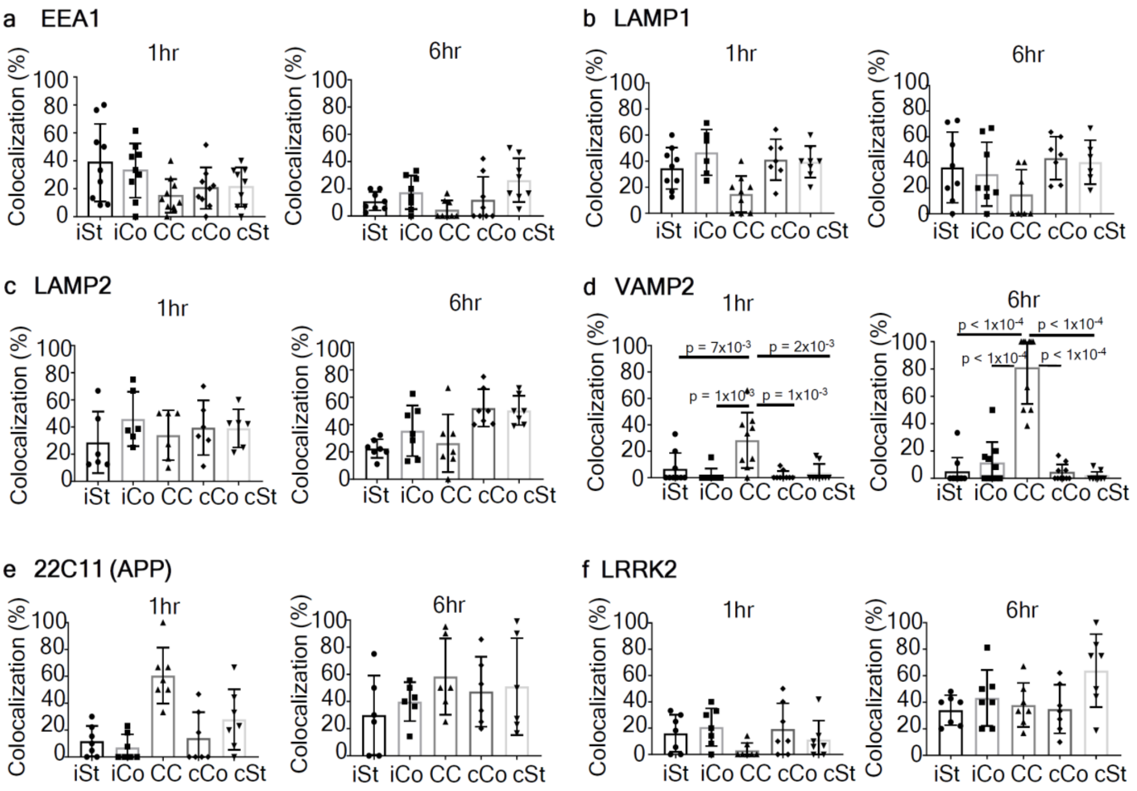

Supplemental Figure S3 Quantitative analysis for Figure 1

Ratio of QD-α-syn-seeds labeled with each marker. Colocalized α-syn number (indicated by white dots) was divided by the number of α-syn positive (pink + white) shown in Fig.1. **a**: EEA1, **b**: LAMP1, **c**: LAMP2, **d**: VAMP2, **e**:22C11, **f**: LRRK2. n =6-9 mice. $p < 0.05$: statistical significance by one-way ANOVA followed by Tukey's post test.

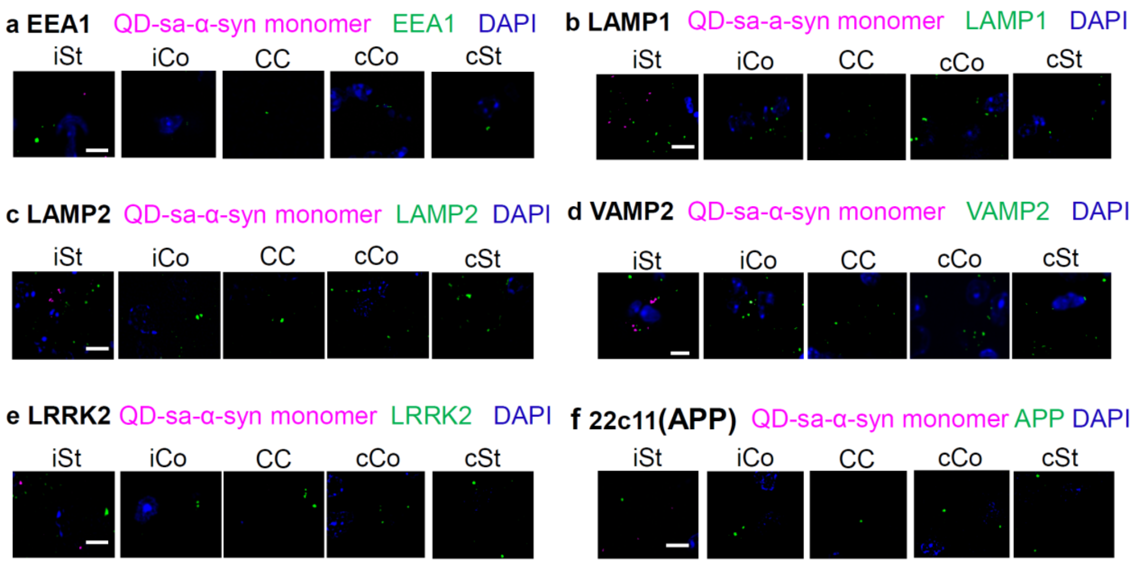

Supplemental Figure S4 Characterization of QD-labeled α-syn monomer

Biotin-tagged α-syn monomer was labeled by streptoavidin-QD (QD-sa-α-syn monomer). QD-sa-α-syn monomer was injected to mice striatum and frozen sections (10μm thick) were immunostained. **(a)** QD-sa-α-syn monomer (pink) and anti-EEA1 immunoreactivity (green). Blue: DAPI. **(b)-(f)**, Other markers (green) : LAMP1**(b)**, LAMP2**(c)**, VAMP2**(d)**, 22C11 for APP**(e)**, LRRK2**(f)**. QD-sa-α-syn monomers were found only in iSt region (injected site) and these QD-sa-α-syn monomer signals show no colocalization with markers. Bar: 5μm.

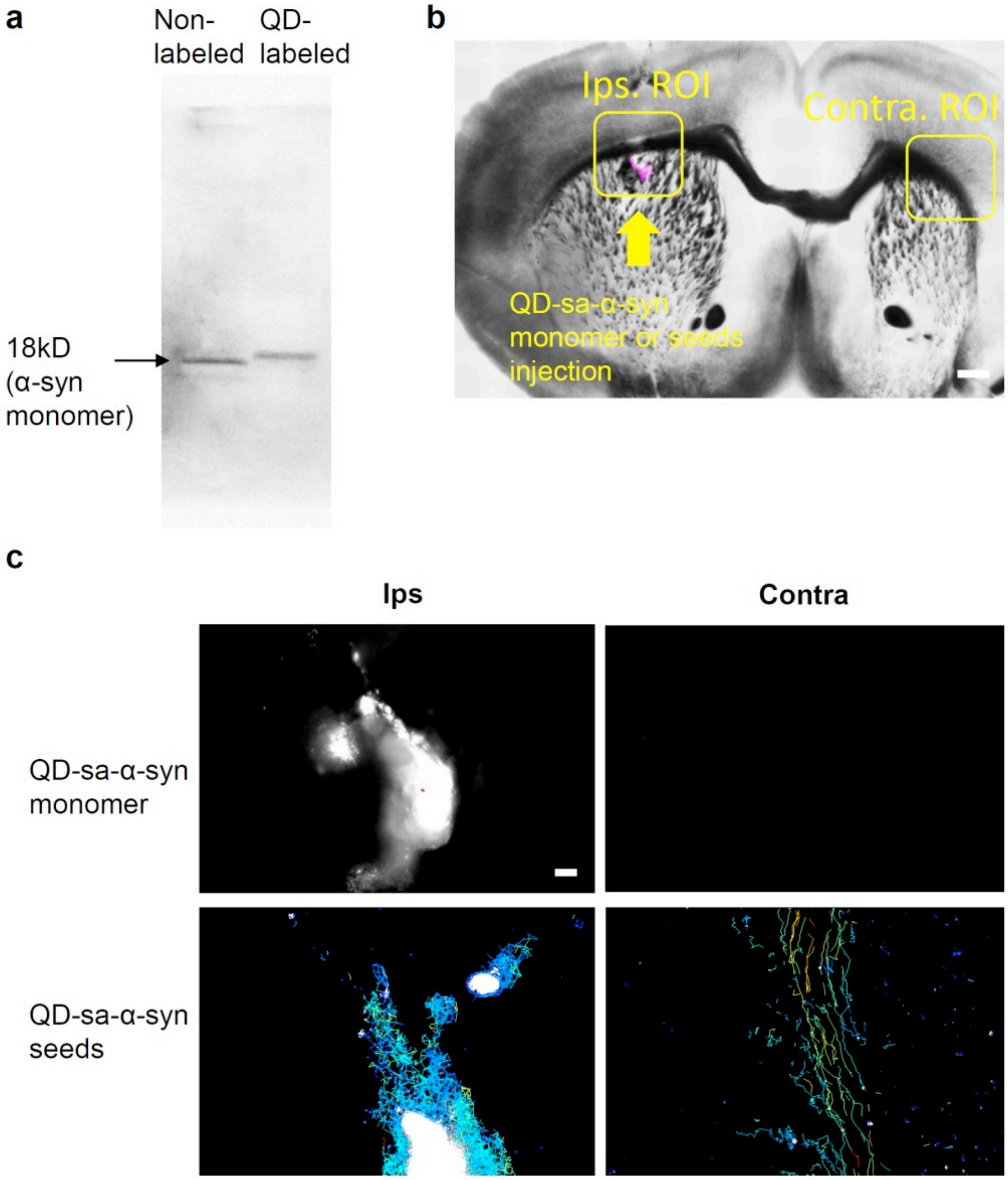

Supplemental Figure S5 QD-labeled α-syn monomer does not show spreading to the contralateral hemisphere.

(a) Immunoblotting of non-label α-syn monomer (left) and QD-labeled α-syn monomer (QD-sa-α-syn monomer, right). Arrow: 18kD. **(b)** ROI of Ips (area around QD-sa-α-syn monomer or QD-sa-α-syn seed injection site) and Contra. QD- sa-α-syn seeds were prepared by conjugating biotin-tagged a-syn seeds and streptoavidin–QD. Bar:500μm. **(c)** Tracking analysis of QD- sa-α-syn monomer (upper panels) and QD- sa-α-syn seeds (lower panels). Left panels: Ips, Right panels: Contra. Bar:10μm. No movement of QD-

sa-α-syn monomer signals (upper panel:Ips) and no signal of QD- sa-α-syn monomer (upper panel:Contra) was observed. On the other hand, the movements of QD- sa-α-syn seeds (lower panel) in Ips and Contra were observed.

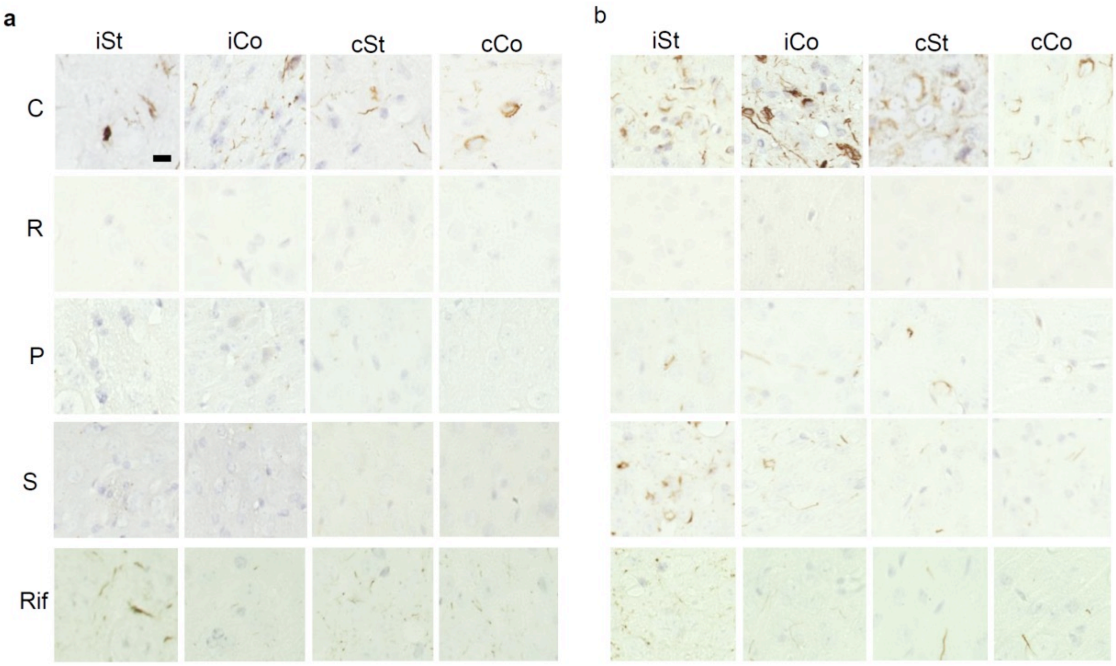

Supplemental Figure S6 Higher magnification of Fig. 5g and h

Higher magnification of Fig. 5g (a) and 5h (b). Bar: 5μm.

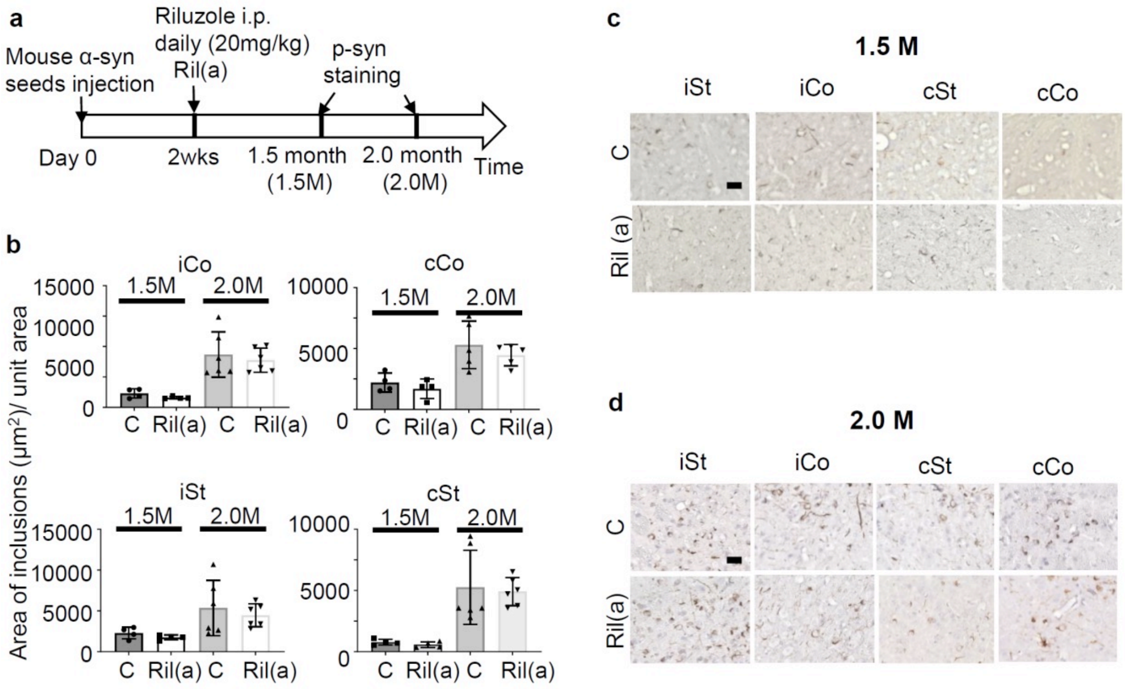

Supplemental Figure S7 No effect of Riluzole administered from 2weeks after α-syn seed injection

(a) Experimental procedure for riluzole effects on phosphorylated α-syn (p-syn) pathology after mouse α-syn seed injection. Riluzole was administered from 2weeks after mouse α-syn seed injection. **(b, c, d)** No difference of inclusions area was observed between control(C)(without riluzole treatment) and Ril(a). Bar: 10μm.

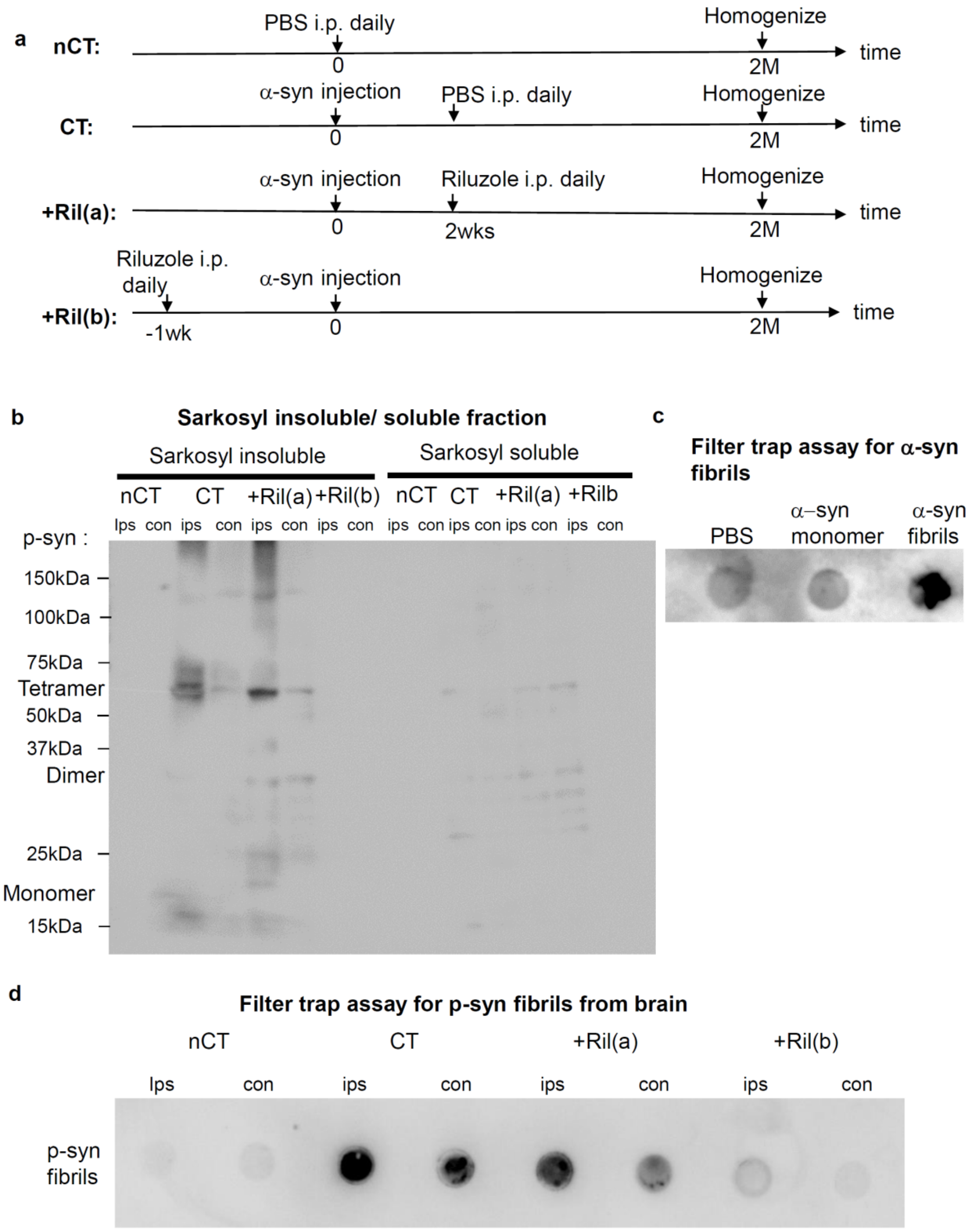

Supplemental Figure S8 Pathological p-syn aggregates in sarkosyl insoluble fraction and their propagation prevented by pre-administration of riluzole in mouse brains

(**a**) Experimental procedures. nCT: PBS i.p., CT: PBS i.p. after mouse α-syn injection, +Ril (a) riluzole i.p. daily from 2 weeks after mouse α-syn injection, +Ril (b) riluzole i.p. daily from one week before mouse α-syn injection. (**b**) Immunoblotting of sarkosyl insoluble fractions (left) and sarkosyl soluble fractions (right). High level of pathological p-syn aggregates in the sarkosyl insoluble fractions of CT and +Ril (a) brains. (**c**) Filter trap assay of PBS (left), α-syn monomer (middle) and α-syn fibrils (right). α-syn immunoreactivity(LB509) was observed only for α-syn fibrils. (**d**) Filter trap assay of Ips and Con brain regions from nCT, CT, +Ril (a), +Ril (b) mice. Increased p-syn immunoreactivities were observed for CT and +Ril (a).

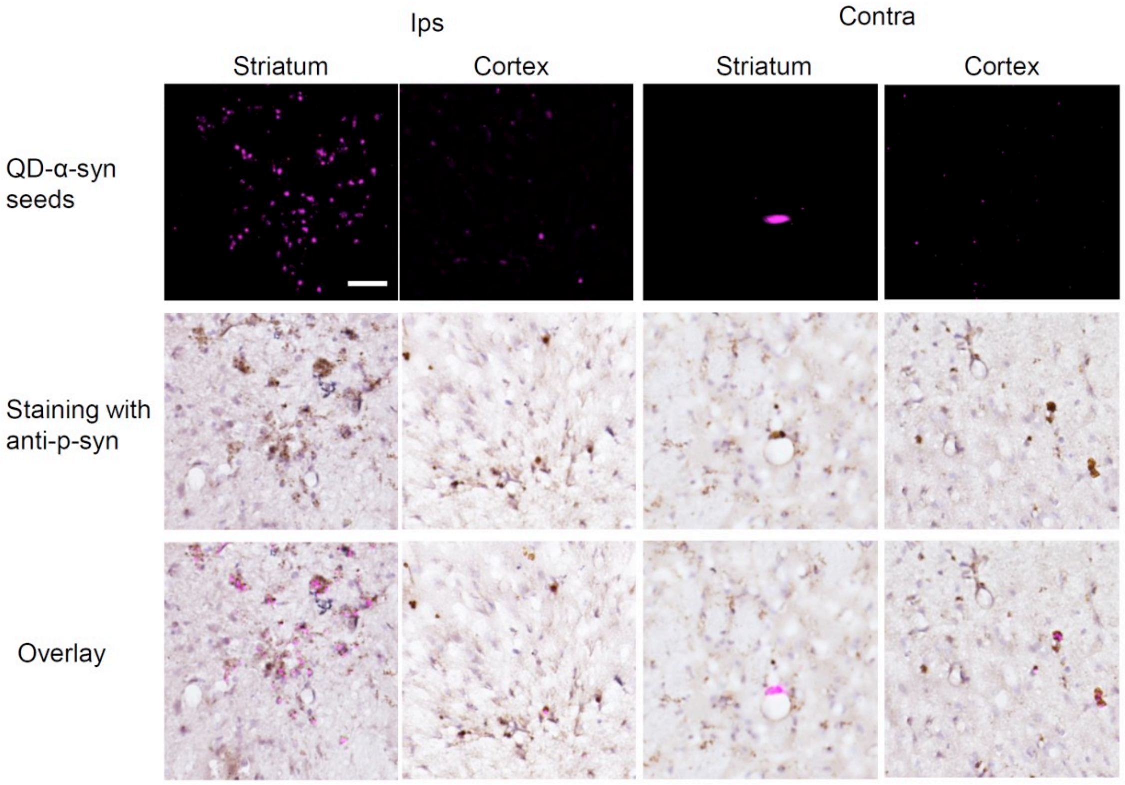

Supplemental Figure S9 Pathological p-syn immunoreactivity at 2 months after QD-a-syn seeds injection

QD-a-syn-seeds were injected to the ipsilateral (Ips) striatum and seed spreading toward Ips cortex, contralateral (Contra) striatum and cortex. Each ROI was stained with anti-p-syn antibody (DAB), revealing the p-syn deposits in the QD-a-syn-seeds injected mice. Bar: 10um

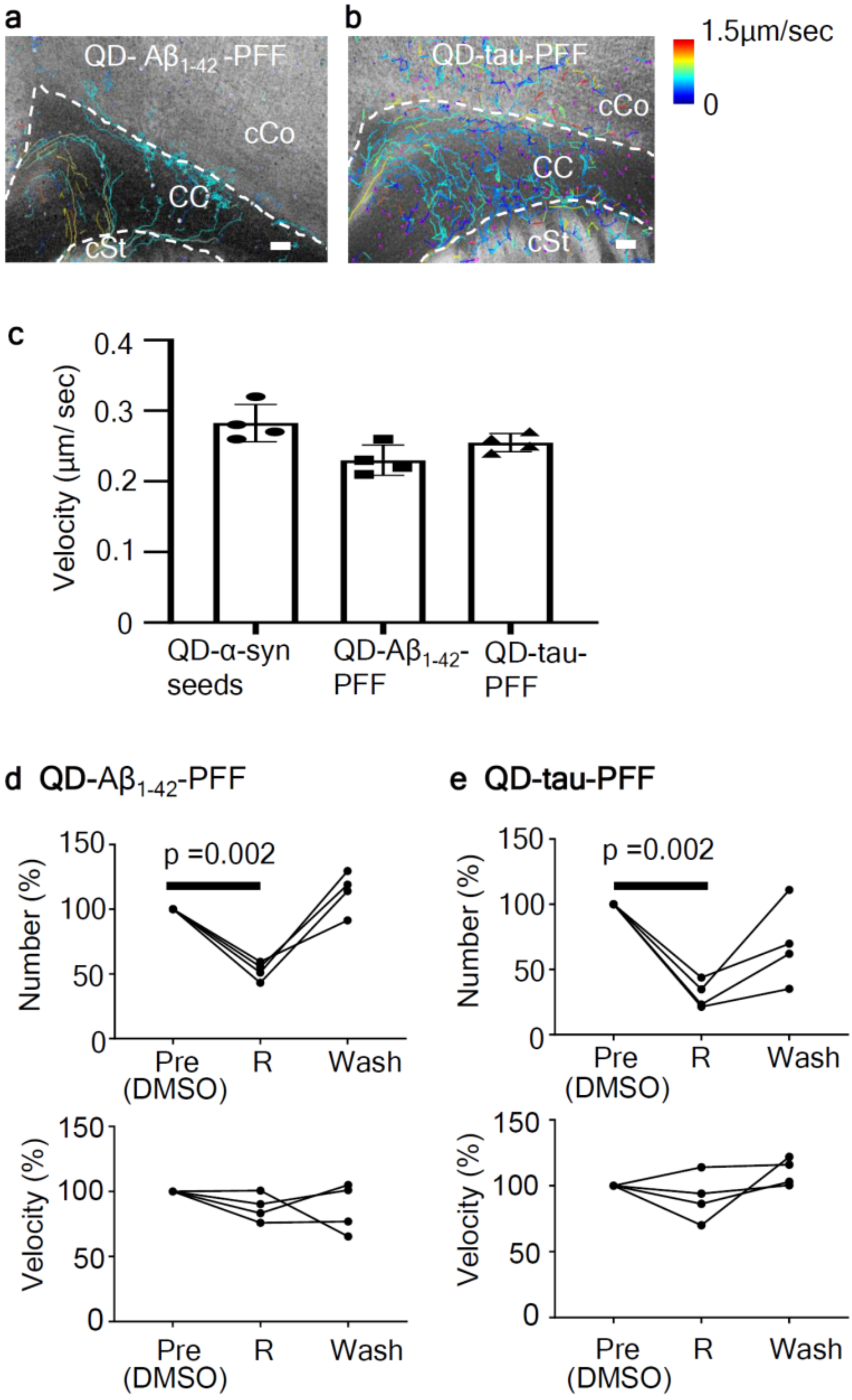
a
QD- Aβ1-42 -PFF
cCo
CC
cSt
b
QD-tau-PFF
cCo
CC
cSt
1.5µm/sec
0
c
Velocity (µm/ sec)
0.4
0.3
0.2
0.1
0
QD-α-syn seeds
QD-Aβ1-42-PFF
QD-tau-PFF
d QD-Aβ1-42-PFF
e QD-tau-PFF
Number (%)
Velocity (%)
150
100
50
0
p =0.002
Pre (DMSO)
R
Wash

Supplemental Figure S10 Dynamics of Aβ_{1-42}-PFFs and tau-PFFs labeled with QD

(a, b) QD- Aβ_{1-42}-PFFs and QD-tau-PFFs dynamics were analyzed in contralateral corpus callosum (cc), cCo and cSt as conducted for α-syn seeds. Bar: 50μm. Tracking was conducted by Image-J Trackmate. **c** QD-α-syn seeds, QD- Aβ_{1-42}-PFFs and QD-tau-PFFs showed similar velocity. Reduction of migrating numbers of QD- Aβ_{1-42}-PFF (**d**) and QD-tau-PFF (**e**) were observed by riluzole treatment. n = 4 mice. $p < 0.05$: statistical significance by one-way ANOVA followed by Tukey's post test.

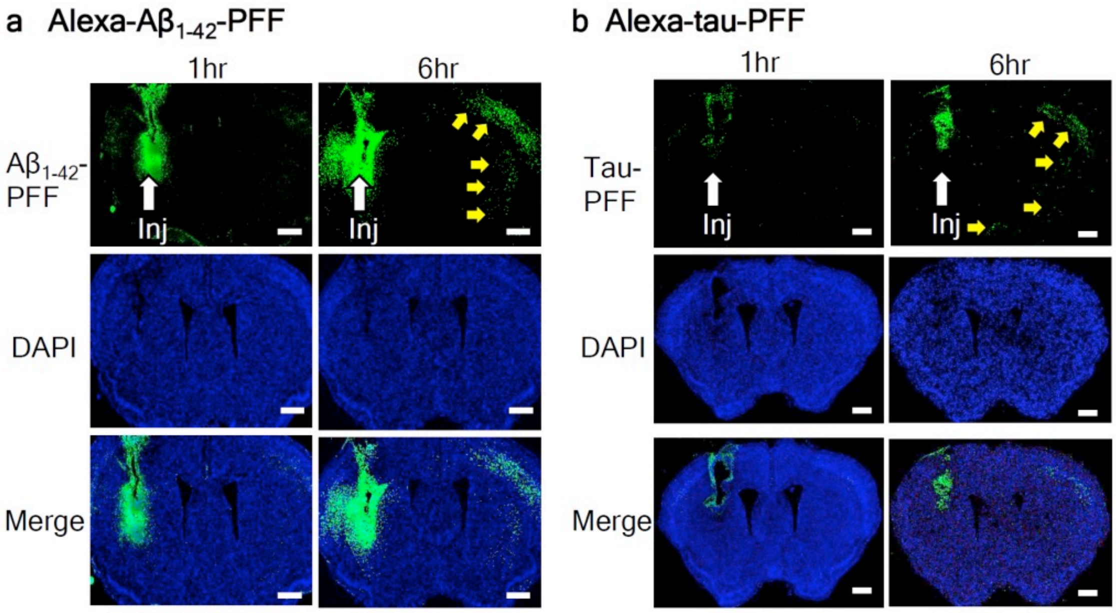

Supplemental Figure S11 Dissemination of Alexa488- PFFs

Alexa488-labeled human $A\beta_{1\text{-}42}$ -PFFs (**a**), and tau-PFFs (**b**) in the coronal 10µm thick section of mouse brain. PFFs were injected at striatum. Left panels: 1hr after injection. Right panels: 6hr after injection. Yellow arrows: disseminated Alexa488-PFFs. Scale bar: 500µm. n = 3 mice.

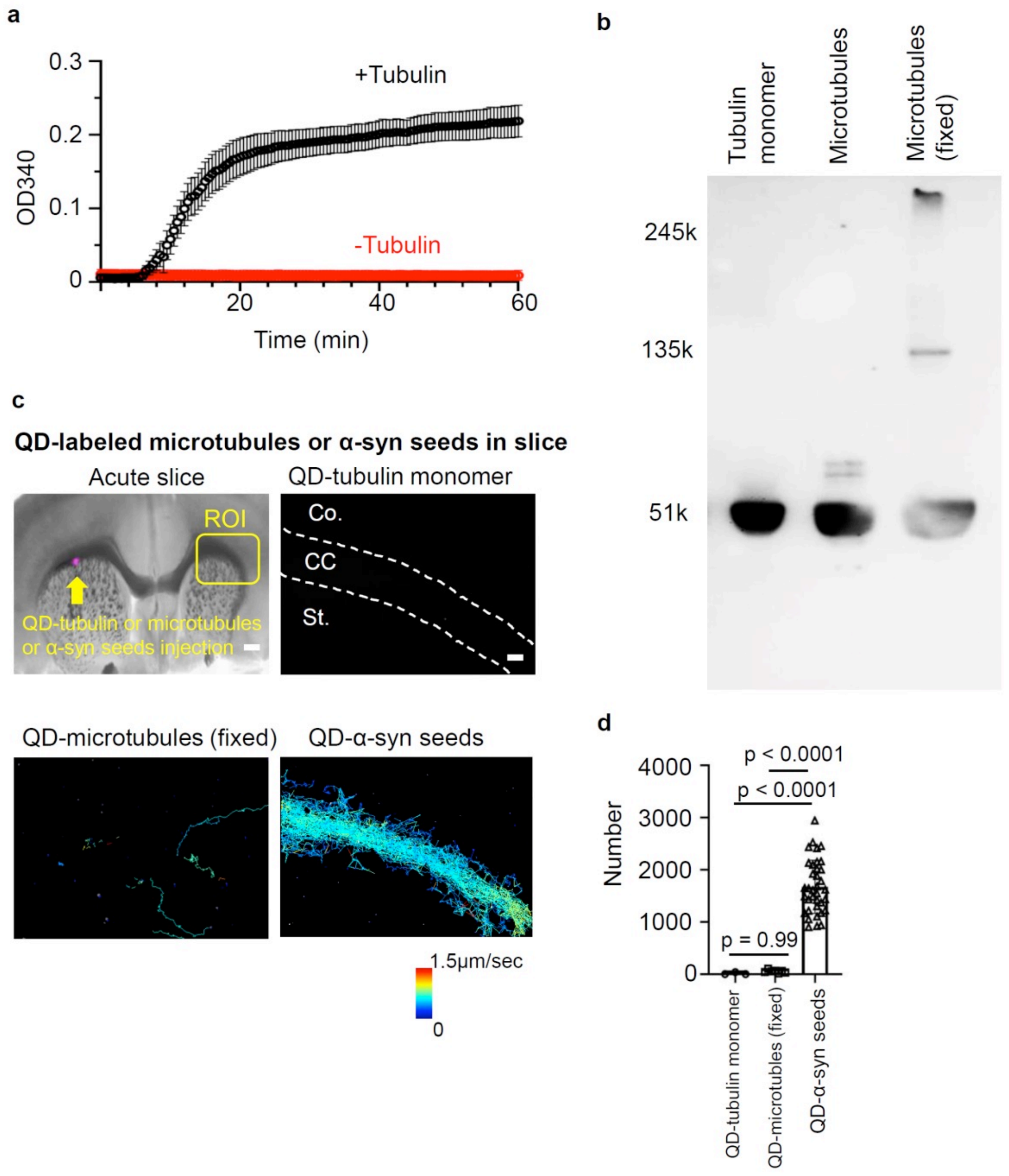

Supplemental Figure S12 No migration of QD-microtubules in living slice

(**a**) Polymerization of tubulin (5mg/mL) was monitored at OD_{340} by SpectraMax M2. Black: Tubulin polymerization, red: same reaction without tubulin as negative control. Number of samples = 6. (**b**) Western blot analysis for tubulin. Immunoreactive band was detected by anti-beta-tubulin. (**c**) Very few migration signals of QD-labeled microtubules in living slice were observed. Tubulin monomer and microtubules were labeled with QD as α-syn seeds. Tracking analyses in ROI were shown. Co.: cortex, CC: corpus callosum, St.: striatum. Bar: 500μm (left panel), 50μm (upper right and

bottom panels). (**d**) Statistical analyses for migration number. Mean values for the migration numbers were calculated. Number of mice: N=3 (QD-tubulin monomer), 7 (QD-microtubules (fixed)), 35 (QD-α-syn seeds).
