# Supplementary material for: Quantum-dot-labeled synuclein seed assay identifies drugs modulating the experimental prion-like transmission
Source: Commun Biol. 2022 Jun 29;5:636. doi: 10.1038/s42003-022-03590-8 (PMC9243017; doi:10.1038/s42003-022-03590-8)
Supplement: Supplementary file 3 — Description of Additional Supplementary Files [file 42003_2022_3590_MOESM3_ESM.pdf]

Description of Additional Supplementary Files

File name: Supplemental data 1
Description: The source data of Fig 2d

File name: Supplemental data 2
Description: The source data of Fig 3b-3h

File name: Supplemental data 3
Description: The source data of Fig4a-f

File name: Supplemental data 4
Description: The source data of Fig5a-f

File name: Supplemental data 5
Description: The source data of Supplemental fig s1c and e

File name: Supplemental data 6
Description: The source data of supplemental figS2

File name: Supplemental data 7
Description: The source data of supplemental figS3a-f

File name: Supplemental data 8
Description: The source data of supplemental figs7b

File name: Supplemental data 9
Description: The source data of supplemental figs8b

File name: Supplemental data 10
Description: The source data of supplemental figs10c-e

File name: Supplemental data 11
Description: The sourse data of supplemental figs12

File name: Supplemental movie 1

Description: QD-α-syn seed dynamics in the slice

File name: Supplemental movie 2

Description: Reduction of QD-α-syn seed dynamics in the slice by clathrin-dependent endocytosis inhibitor (dynasore)

File name: Supplemental movie 3

Description: Retrograde transport of QD-α-syn seeds

File name: Supplemental movie 4

Description: Riluzole reduces QD-α-syn seeds
