# Supplementary material for: Quantum-dot-labeled synuclein seed assay identifies drugs modulating the experimental prion-like transmission
Source: Commun Biol. 2022 Jun 29;5:636. doi: 10.1038/s42003-022-03590-8 (PMC9243017; doi:10.1038/s42003-022-03590-8)
Supplement: Supplementary file 8 — Reporting Summary [file 42003_2022_3590_MOESM8_ESM.pdf]

nature portfolio

Corresponding author(s): Nobuyuki Nukina

Last updated by author(s): 28/ Feb/ 2022

Reporting Summary

Nature Portfolio wishes to improve the reproducibility of the work that we publish. This form provides structure for consistency and transparency in reporting. For further information on Nature Portfolio policies, see our Editorial Policies and the Editorial Policy Checklist.

Statistics

For all statistical analyses, confirm that the following items are present in the figure legend, table legend, main text, or Methods section.

n/a	Confirmed	
☐	☒	The exact sample size (*n*) for each experimental group/condition, given as a discrete number and unit of measurement
☐	☒	A statement on whether measurements were taken from distinct samples or whether the same sample was measured repeatedly
☐	☒	The statistical test(s) used AND whether they are one- or two-sided<br>*Only common tests should be described solely by name; describe more complex techniques in the Methods section.*
☐	☒	A description of all covariates tested
☐	☒	A description of any assumptions or corrections, such as tests of normality and adjustment for multiple comparisons
☐	☒	A full description of the statistical parameters including central tendency (e.g. means) or other basic estimates (e.g. regression coefficient) AND variation (e.g. standard deviation) or associated estimates of uncertainty (e.g. confidence intervals)
☒	☐	For null hypothesis testing, the test statistic (e.g. *F*, *t*, *r*) with confidence intervals, effect sizes, degrees of freedom and *P* value noted<br>*Give P values as exact values whenever suitable.*
☒	☐	For Bayesian analysis, information on the choice of priors and Markov chain Monte Carlo settings
☒	☐	For hierarchical and complex designs, identification of the appropriate level for tests and full reporting of outcomes
☒	☐	Estimates of effect sizes (e.g. Cohen's *d*, Pearson's *r*), indicating how they were calculated

Our web collection on statistics for biologists contains articles on many of the points above.

Software and code

Policy information about availability of computer code

Data collection	See below for full description of collection software.<br>Imaging and immunohistochemistry：BZX-viewer(Keyence, Osaka, Japan).<br>Electron microscopy: EMIP-SP (Hitachi, Tokyo, Japan)
Data analysis	Collected imaging data was analyzed by BZX-analyzer (Keyence) and saved as time lapse data. The time lapse data was analyzed by Image-J software and movement of amyloids-PFF were analyzed by trackmate plug-in. The saved data for tracking amyloids-PFF in excel file were analyzed by Graph Pad Prism v. 7.00 to determine the statistical significant difference.

For manuscripts utilizing custom algorithms or software that are central to the research but not yet described in published literature, software must be made available to editors and reviewers. We strongly encourage code deposition in a community repository (e.g. GitHub). See the Nature Portfolio guidelines for submitting code & software for further information.

Data

Policy information about availability of data

All manuscripts must include a data availability statement. This statement should provide the following information, where applicable:

- Accession codes, unique identifiers, or web links for publicly available datasets
- A description of any restrictions on data availability
- For clinical datasets or third party data, please ensure that the statement adheres to our policy

The data that support the findings of this study are available from corresponding author, upon reasonable request.

Field-specific reporting

Please select the one below that is the best fit for your research. If you are not sure, read the appropriate sections before making your selection.

☒ Life sciences ☐ Behavioural & social sciences ☐ Ecological, evolutionary & environmental sciences

For a reference copy of the document with all sections, see nature.com/documents/nr-reporting-summary-flat.pdf

Life sciences study design

All studies must disclose on these points even when the disclosure is negative.

Sample size	No formal sample size calculation was performed for samples. For mouse experiments, sample sizes (3-4 mice) were rationalized by balancing sufficient replication to detect moderate effect sizes with reduction of total animals used.
Data exclusions	Recording data which get out of focus during recording(e.g. drift of slices) were excluded.
Replication	All experiments were repeated at least three independent differentiations. Data for all attempts of the replication were successful was applied to data storage and analysis.
Randomization	All experiments were differentiated and analyzed together in each experiment.
Blinding	Investigators were blinded to group allocation for the drugs used in imaging and immunostaining experiments.

Reporting for specific materials, systems and methods

We require information from authors about some types of materials, experimental systems and methods used in many studies. Here, indicate whether each material, system or method listed is relevant to your study. If you are not sure if a list item applies to your research, read the appropriate section before selecting a response.

Materials & experimental systems

n/a	Involved in the study	
☐	☒	Antibodies
☐	☒	Eukaryotic cell lines
☒	☐	Palaeontology and archaeology
☐	☒	Animals and other organisms
☒	☐	Human research participants
☒	☐	Clinical data
☒	☐	Dual use research of concern

Methods

n/a	Involved in the study	
☒	☐	ChIP-seq
☒	☐	Flow cytometry
☒	☐	MRI-based neuroimaging

Antibodies

Antibodies used	We used primary antibodies and secondary antibodies. Antibodies used were as follows: 1) primary antibodies: anti-α-syn(mouse monoclonal, LB509, 1:100, Invitrogen, MD, USA) for acute α-syn seeds, vesicle-associated membrane protein (vamp) 2 (mouse monoclonal, 104211, 1:100, Synaptic systems Gottingen Germany) for vesicle-associated membrane protein, EEA1 (mouse monoclonal, E41120, 1:100, Transduction laboratories Null USA) for early endosome, LAMP1 (mouse monoclonal, 09671D, 1:100, Pharmingen Franklin Lakes NJ USA) for lysosomal-associated membrane protein 1, LAMP2 (mouse monoclonal, 558756, 1:100, Pharmingen) for lysosomal-associated membrane protein 2, LRRK2 (rabbit monoclonal, 3514-1, 1:100, Epitomics Balingen CA USA) for leucine rich repeat kinase 2, 22C11 (mouse monoclonal, MAB-348, 1:100, Chemicon Bilerica MA USA) for amyloid precursor protein, anti-DARPP32 (rabbit polyclonal, AB10518, 1:100, Millipore, MA, USA), anti-tyrosine hydroxylase (mouse monoclonal, MAB-318, 1:100, Milipore, MA, USA). 2) secondary antibodies: Alexafluor 488 highly cross-absorbed (goat anti-mouse or goat anti-rabbit, A11029 or A11034, 1:100, Invitrogen MD USA) .
Validation	All antibodies were selected based on validation statements on the manufacture's brochure and website for reactivity to protein and species specified by the manufacturer.

Eukaryotic cell lines

Policy information about cell lines

Cell line source(s)	Neuro2a cells
Authentication	The cell lines were not be authenticated.

Mycoplasma contamination	Mycoplasma was tested and contamination was negative.
Commonly misidentified lines (See ICLAC register)	None

Animals and other organisms

Policy information about studies involving animals; ARRIVE guidelines recommended for reporting animal research

Laboratory animals	Mice- C57BL6J(B6J) male mice age 8weeks old were purchased from Shimizu animal supply (Kyoto, Japan).
Wild animals	The study did not involve wild animals
Field-collected samples	The study did not involve the use of field-collected samples.
Ethics oversight	Performed under Ethical approval for animal research obtained by Doshisha University (Kyoto, Japan).

Note that full information on the approval of the study protocol must also be provided in the manuscript.
